# Childhood cancer survival trends in Queensland 1956-80.

**DOI:** 10.1038/bjc.1984.79

**Published:** 1984-04

**Authors:** W. R. McWhirter, V. Siskind

## Abstract

The true survival rates for the various forms of childhood cancer are best determined from a population-based study rather than from the results of clinical trials. Population-based survival rates have been calculated for four periods between 1956 and 1980 in Queensland. There was a significant improvement in survival for children who developed cancer after 1973 compared with those diagnosed before this date. There has however been no significant improvement in the survival rate for childhood cancer overall, or for acute lymphoblastic leukaemia since 1973. Over the 25 year period significant trends in survival rates were seen in acute lymphoblastic leukaemia, non-Hodgkin's lymphoma, Hodgkin's disease, Wilms' tumour, medulloblastoma, and retinoblastoma. No such trend was seen for acute non-lymphoblastic leukaemia, neuroblastoma, rhabdomyosarcoma, juvenile or anaplastic astrocytoma, brain stem glioma, histiocytosis X, or bone tumours. There is a need for continuing research into better methods of treatment of childhood cancer.


*Br. J. Cancer* (1984), **49**, 513–519

### Childhood cancer survival trends in Queensland 1956–80

W.R. McWhirter[1] & V. Siskind

[1]*Department of Child Health,* [2]*Department of Social and Preventive Medicine; University of Queensland, Herston Road, Herston, Queensland, Australia.*

**Summary** The true survival rates for the various forms of childhood cancer are best determined from a population-based study rather than from the results of clinical trials. Population-based survival rates have been calculated for four periods between 1956 and 1980 in Queensland. There was a significant improvement in survival for children who developed cancer after 1973 compared with those diagnosed before this date. There has however been no significant improvement in the survival rate for childhood cancer overall, or for acute lymphoblastic leukaemia since 1973. Over the 25 year period significant trends in survival rates were seen in acute lymphoblastic leukaemia, non-Hodgkin's lymphoma, Hodgkin's disease, Wilms' tumour, medulloblastoma, and retinoblastoma. No such trend was seen for acute non-lymphoblastic leukaemia, neuroblastoma, rhabdomyosarcoma, juvenile or anaplastic astrocytoma, brain stem glioma, histiocytosis X, or bone tumours. There is a need for continuing research into better methods of treatment of childhood cancer.

Cancer is an important cause of mortality in children. In the 1–14 year age group it comes second only to accidents as a cause of death. There have been many changes in the management of both leukaemias and solid tumours in the past 30 years. Many of these have been reported, together with results, in the form of clinical trials. Nearly all such trials however require some degree of selection of cases and very little information is available on the survival rates of unselected series of patients, particularly for the most recent years. One large study has been published on childhood cancer survival in Great Britain (Draper *et al.*, 1982), but only includes children who were diagnosed before 1975. Another series from the United States included only white children from the years 1955–71 (Myers *et al.*, 1975). The present study examines the trends in survival rates of childhood cancer in Queensland during the 25 year period from 1956 to 1980.

### Methods

Information on cases was obtained from the Queensland Childhood Malignancy Registry (McWhirter & Bacon, 1981). This is a population based registry which includes all children with malignant tumours and also all intracranial tumours who were under the age of 15 years and resident in Queensland at the date of incidence. Although registration is believed to be complete from 1973 onwards, it is certainly incomplete for the period 1956–72. Registration was retrospective for the earlier period and was obtained from the records of the two children's hospitals in Queensland, from the pathology departments of the Royal Brisbane and Mater Hospitals in Brisbane, and from the Queensland Radium Institute, the centre for radiotherapy for this state. Death certificates were not used as a source of ascertainment. A total of 1,030 patients were registered during the 25 year period 1956–80.

Follow-up information is obtained mainly by the use of questionnaires which are sent annually for the first 5 years, then at 7, 10 and 15 years to the doctor or clinic believed to be caring for the patient. Many of the cases from the earlier years of this study have been followed up by the Queensland Radium Institute which has had an effective information system in operation for many years. In a few cases dates of death were obtained from the Registrar-General. The cases were divided into four groups according to their date of presentation. The period of incomplete registration was divided into two, so as to produce two groups of approximately equal size; Group A contained 202 patients from the period 1956–66, and Group B 249 patients from the period 1967–72. The patients from 1973 onwards were similarly allocated to two groups; Group C (1973–76) contained 252 patients, and Group D (1977–80) 275 patients. Survival data was examined using standard methods of life table analysis (Peto *et al.*, 1977). The *P* values in Table V are based on 3 degrees of freedom for heterogeneity (2 for histiocytosis X because there were no cases of this tumour in Group A), and on one degree of freedom for trend.

Almost all the cases were seen at one or other of the major centres in Brisbane, at least for initial

Correspondence: W.R. McWhirter

Received 19 September 1983; accepted 14 January 1984.

© The Macmillan Press Ltd., 1984

assessment and treatment planning. Because of the vast distances in this state, however, some of the chemotherapy was given, under supervision from the major centre, by the local doctor or at the local hospital.

### Results

The number of cases in each group and the percentage on whom at least some follow-up information was available are shown in Table I. Table II shows the numbers of cases of each of the tumour types studied in the four groups. Tables III and IV give the survival rates at 1, 3, and 5 years, and, for Groups A and B, at 10 years. No cases of neuroblastoma, ependymoma, or histiocytosis X in Group D were yet at risk at 5 years from the date of incidence so that 5-year survival rates could not be calculated for them. The marked improvement in survival rates for childhood cancer overall occurred entirely during the 10-year period from 1967 to 1976. There was no difference in survival between Groups A and B ($\chi^2=0.65$, $P>0.4$), or between Groups C and D ($\chi^2=0.08$, $P>0.7$). Nevertheless the $\chi^2$ values for both heterogeneity and trend are highly significant (Table V). Stratification by diagnostic group made no appreciable difference; the corresponding values for $\chi^2$ after stratification were 64.4 and 54.7.

**Table I** Numbers of cases and proportion followed up

| *Group* | *Years of presentation* | *No. cases* | *% followed up* |
|---|---|---|---|
| A | 1956–66 | 232 | 87.1 |
| B | 1967–72 | 270 | 92.2 |
| C | 1973–76 | 253 | 98.0 |
| D | 1977–80 | 275 | 98.5 |

**Table II** Numbers of cases by diagnosis

| *Group* | *Number of cases in each group* A | *B* | *C* | *D* |
|---|---|---|---|---|
| ALL | 45 | 61 | 72 | 74 |
| ANLL | 6 | 11 | 6 | 14 |
| Non-Hodgkin's lymphoma | 10 | 22 | 12 | 20 |
| Hodgkin's disease | 1 | 8 | 17 | 11 |
| Neuroblastoma | 18 | 16 | 20 | 18 |
| Rhabdomyosarcoma | 5 | 3 | 5 | 13 |
| Wilms' tumour | 18 | 12 | 18 | 17 |
| Juvenile astrocytoma | 24 | 16 | 9 | 8 |
| Anaplastic astrocytoma | 8 | 8 | 4 | 11 |
| Other astrocytoma | 4 | 8 | 8 | 2 |
| Medulloblastoma | 5 | 8 | 13 | 11 |
| Ependymoma | 6 | 8 | 4 | 4 |
| Brain stem glioma | 10 | 13 | 7 | 6 |
| Retinoblastoma | 1 | 8 | 12 | 9 |
| Bone tumours | 15 | 15 | 11 | 11 |
| Histiocytosis X | 0 | 3 | 5 | 10 |
| All Neoplasms | 202 | 249 | 252 | 275 |

**Table III** Survival rates for selected malignancies 1956–72

| *Disease* | *Group A (1956–66)* 1y | *3y* | *5y* | *10y* | *Group B (1967–72)* 1y | *3y* | *5y* | *10y* |
|---|---|---|---|---|---|---|---|---|
| ALL | 33.3 | 8.9 | 2.2 | 0.0 | 68.9 | 14.8 | 6.6 | 4.9 |
| ANLL | 16.7 | 0.0 | 0.0 | 0.0 | 27.3 | 0.0 | 0.0 | 0.0 |
| Non-Hodgkin's lymphoma | 40.0 | 30.0 | 30.0 | 30.0 | 36.4 | 13.6 | 13.6 | 13.6 |
| Hodgkin's disease | 100.0 | 100.0 | 100.0 | 100.0 | 87.5 | 62.5 | 37.5 | 37.5 |
| Neuroblastoma | 38.9 | 22.2 | 22.2 | 22.2 | 37.5 | 31.3 | 31.3 | 31.3 |
| Rhabdomyosarcoma | 20.0 | 20.0 | 20.0 | 20.0 | 66.7 | 66.7 | 66.7 | 66.7 |
| Wilms' tumour | 61.1 | 38.9 | 38.9 | 38.9 | 75.0 | 75.0 | 75.0 | 75.0 |
| Juvenile astrocytoma | 91.7 | 79.2 | 79.2 | 79.2 | 81.3 | 62.5 | 62.5 | 56.3 |
| Anaplastic astrocytoma | 25.0 | 12.5 | 12.5 | 12.5 | 75.0 | 50.0 | 37.5 | 37.5 |
| Other astrocytoma | 75.0 | 75.0 | 75.0 | 25.0 | 87.5 | 75.0 | 75.0 | 62.5 |
| Medulloblastoma | 60.0 | 20.0 | 20.0 | 20.0 | 25.0 | 12.5 | 12.5 | 12.5 |
| Ependymoma | 66.7 | 50.0 | 50.0 | 50.0 | 87.5 | 37.5 | 25.0 | 25.0 |
| Brain stem glioma | 40.0 | 30.0 | 30.0 | 30.0 | 23.1 | 7.7 | 7.7 | 7.7 |
| Retinoblastoma | 100.0 | 0.0 | 0.0 | 0.0 | 100.0 | 87.5 | 87.5 | 87.5 |
| Bone tumours | 60.0 | 40.0 | 40.0 | 33.3 | 53.3 | 26.7 | 13.3 | 13.3 |
| Histiocytosis X | — | — | — | — | 33.3 | 33.3 | 33.3 | 33.3 |
| All tumours | 50.0 | 33.2 | 31.2 | 28.7 | 60.6 | 34.5 | 29.7 | 28.5 |

**Table IV** Survival rates for selected malignancies 1973–80

| *Disease* | *Group C (1973–76)* | | | *Group D (1977–80)* | | |
|---|---|---|---|---|---|---|
| | *1y* | *3y* | *5y* | *1y* | *3y* | *5y* |
| ALL | 81.9 | 58.3 | 45.8 | 82.4 | 61.6 | 47.9 |
| ANLL | 66.7 | 16.7 | 16.7 | 57.1 | 19.1 | 0.0 |
| Non-Hodgkin's lymphoma | 83.3 | 75.0 | 58.3 | 65.0 | 60.0 | 60.0 |
| Hodgkin's disease | 100.0 | 100.0 | 100.0 | 100.0 | 100.0 | 100.0 |
| Neuroblastoma | 55.0 | 35.0 | 30.0 | 50.0 | 22.2 | — |
| Rhabdomyosarcoma | 60.0 | 20.0 | 20.0 | 53.9 | 46.2 | 46.2 |
| Wilms' tumour | 72.2 | 66.7 | 61.1 | 94.1 | 80.9 | 80.9 |
| Juvenile astrocytoma | 77.8 | 77.8 | 77.8 | 100.0 | 72.9 | 72.9 |
| Anaplastic astrocytoma | 25.0 | 25.0 | 25.0 | 63.6 | 27.3 | 27.3 |
| Other astrocytoma | 87.5 | 62.5 | 62.5 | 100.0 | 50.0 | 50.0 |
| Medulloblastoma | 69.2 | 46.2 | 38.5 | 90.9 | 63.6 | 63.6 |
| Ependymoma | 100.0 | 25.0 | 25.0 | 100.0 | — | — |
| Brain stem glioma | 28.9 | 0.0 | 0.0 | 50.0 | 0.0 | 0.0 |
| Retinoblastoma | 91.7 | 91.7 | 91.7 | 100.0 | 100.0 | 100.0 |
| Bone tumours | 100.0 | 54.6 | 36.4 | 90.9 | 52.5 | 52.5 |
| Histiocytosis X | 100.0 | 100.0 | 100.0 | 80.0 | 70.0 | — |
| All tumours | 77.4 | 59.5 | 53.2 | 76.4 | 57.1 | 52.0 |

**Table V** Survival analysis

| *Disease* | *Chi-squared and P value for Heterogeneity* | | *Trend* | |
|---|---|---|---|---|
| ALL | 85.9856 | <0.0005 | 72.1547 | <0.0005 |
| ANLL | 5.6998 | NS | 3.8016 | NS |
| Non-Hodgkin's lymphoma | 11.3471 | <0.01 | 7.0627 | <0.01 |
| Hodgkin's disease | 22.4815 | <0.0005 | 9.2631 | <0.005 |
| Neuroblastoma | 0.6954 | NS | 0,0334 | NS |
| Rhabdomyosarcoma | 2.2025 | NS | 0.4351 | NS |
| Wilms' tumour | 7.5180 | NS | 5.5249 | <0.025 |
| Juvenile astrocytoma | 1.7989 | NS | 0.0101 | NS |
| Anaplastic astrocytoma | 3.0585 | NS | 0.1695 | NS |
| Other astrocytoma | 1.7716 | NS | 0.0688 | NS |
| Medulloblastoma | 7.2160 | NS | 5.3133 | <0.025 |
| Ependymoma | 0.7905 | NS | 0.2965 | NS |
| Brain stem glioma | 2.0648 | NS | 0.2616 | NS |
| Retinoblastoma | 13.7936 | <0.005 | 4.3022 | <0.05 |
| Bone tumours | 4.2815 | NS | 1.7980 | NS |
| Histiocytosis X | 6.31 | <0.05 | 0.9543 | NS |
| All tumours | 55.2690 | <0.0005 | 46.5241 | <0.0005 |

The greatest improvement in survival occurred in acute lymphoblastic leukaemia (ALL). The 5-year survival rate increased from 2.2% in Group A to 6.6% in Group B (Figure 1) and to 45% in Group C, but only to 48% in Group D (Figure 2). $\chi^2$ for Group C *versus* Group D=0.14, $P>0.7$. Significant trends in survival rates over the 4 groups were also seen in non-Hodgkin's lymphoma, Hodgkin's disease, Wilms' tumour, medulloblastoma, retinoblastoma and bone tumours (Table V).

### Discussion

It is almost certain that some of the patients not registered, or lost to follow-up in the period before 1972 were those who died early, so that survival rates calculated for Groups A and B may be slightly higher than the true values. The number of such patients must however be small. Until recently, specialist treatment was not available for children outside the capital, Brisbane. Cases were therefore

**Figure 1** Survival in ALL, 1956–1972.

**Figure 2** Survival in ALL, 1973–1980.

generally transferred to one of the major hospitals in Brisbane at an early stage in their illness. In interpreting the 5-year survival rate in Group D, it is important to appreciate that this is an estimate made from life-table analysis. Most of the patients in this group have not yet had 5 years of follow-up. The 5-year survival rates for Group D should therefore be regarded as tentative. It was felt nevertheless that they should be included in an attempt to provide up-to-date information.

This study confirms the improvement in survival which has been reported for childhood cancer as a whole as well as for several individual types of tumour (Draper *et al.*, 1982; Myers *et al.*, 1975). The survival rates quoted in these two series may be compared with those in Tables III and IV, and are generally of the same order as those for the present series (see Table VI).

The most important advance, in terms of reduction of overall cancer mortality, has been the increase in the survival rate for ALL. Before 1970 this disease was almost invariably fatal. Almost half the children in Group C survived 5 years from the time of diagnosis, but there was virtually no further gain in survival for those in Group D. The development of carefully planned chemotherapy including multiple drug induction and consolidation regimes, CNS prophylaxis with combined cranial radiation and intrathecal chemotherapy, and better supportive care such as vigorous treatment of actual and potential infection have all contributed to the improvement. For acute non-lymphoblastic leukaemia (ANLL), there has been a considerable increase in the proportion of children attaining an initial remission, but the subsequent relapse rate remains high (Wilbur *et al.*, 1981). As a result, there are still very few long-term survivors.

The introduction of effective chemotherapy, with or without radiotherapy, for non-Hodgkin's lymphoma (Wollner *et al.*, 1976) has greatly improved long-term survival in this condition, and most children who are alive at 4 years from diagnosis are likely to be cured of their disease (Anderson *et al.*, 1983). Current methods of treatment for Hodgkin's disease, including combined chemotherapy and extended field radiotherapy are now producing extremely high cure rates, probably accompanied by less morbidity than was seen with older methods of treatment (Jenkin & Berry, 1980). Despite numerous attempts to improve the prognosis for neuroblastoma, long term results have remained disappointing, especially in older children who tend to have advanced disease at the time of diagnosis.

In contrast with the results which might have been expected from the literature on the use of chemotherapy in rhabdomyosarcoma (Bizer, 1980), there was no significant improvement in the survival rates for this tumour. The number of cases is, however, small. This together with the fact that the different histological criteria may have been applied during the 25 year period must cast slight doubt on the accuracy of the survival rates in Groups A and B. The use of chemotherapy with agents such as vincristine, dactinomycin, and cyclophosphamide has reduced the need for radical surgery, especially in embryonal rhabdomyosarcoma, so that the long term morbidity in the survivors is probably less than previously.

There has been a substantial improvement in the survival rate for Wilms' tumour. This is in keeping with published results (D'Angio *et al.*, 1981) and reflects the universal use of chemotherapy, especially vincristine and dactinomycin, in this tumour. The application of radiotherapy has also been refined, and it is now clear that radiotherapy

**Table VI** 5-year survival rates in Queensland, Great Britain and U.S. whites

| *Tumour type* | *Queensland 1967–72* | *Great Britain 1962–70* | *Manchester 1954–73* | *U.S. whites 1965–69* |
|---|---|---|---|---|
| ALL | 7 | | 12 | 6 |
| Non-Hodgkin's lymphoma | 14 | 20 | 24 | 20 |
| Hodgkin's disease | 38 | 53 | 56 | 66 |
| Neuroblastoma | 31 | 18 | 14 | 22 |
| Rhabdomyosarcoma | 67 | 20 | 21 | 36[a] |
| Wilms' tumour | 75 | 34 | 44 | 60 |
| Juvenile astrocytoma | 63 | | 71 | |
| Medulloblastoma | 13 | 18 | 32 | 32 |
| Ependymoma | 13 | 23 | 25 | |
| Retinoblastoma | 88 | 85 | 83 | 91 |
| Bone tumours | 13 | 22 | 16 | 22 |
| All cancers | 30 | | | 34 |

[a]soft tissue sarcomas.

is not required in cases where the disease is confined to the kidney (D'Angio *et al.*, 1981).

Amongst the brain tumours there is a mixed picture dependent on the morphological diagnosis. There is an obvious difference in the survival pattern between juvenile astrocytomas of low malignancy and the anaplastic variety. This has been noted previously (Draper *et al.*, 1982; Bloom, 1982*a*), and confirms the value of accurate histological diagnosis. In neither type was a significant trend in survival rates observed. Unlike the astrocytomas, there has been a significant improvement in survival for medulloblastoma. Changes in radiotherapy techniques have led to higher cure rates for this tumour (Berry *et al.*, 1981), and adjuvant chemotherapy may be resulting in further improvement, with 5-year survival rates of ~70% being reported (Bloom, 1982*b*). Some of the patients with medulloblastoma in Group D received various forms of chemotherapy in addition to surgery and radiotherapy. Although some of the patients with ependymoma also received chemotherapy, it is not yet possible to assess the effect of this because of the relative rarity of this tumour. In general, the patients with brain stem glioma were treated by radiotherapy alone and the results were extremely poor. Although there were some long-term survivors in Groups A and B, the longest survival amongst Groups C and D was 26 months.

The outlook in retinoblastoma was good in the later 3 groups and survival rates were similar to those in Britain (Draper *et al.*, 1982). The survival rates for bone tumours appear quite high in Groups C and D. Comparison with other series is however difficult since the pattern of incidence is quite different from that in other reported series, Ewing's tumour being much commoner than osteosarcoma in Queensland (McWhirter & Bacon, 1981). The reason for this is not known. In histiocytosis X, there appears to have been some improvement in survival, but the trend was not statistically significant, perhaps because of the small numbers. The possibility also exists that in Group B there may have been some under-ascertainment of cases of localised disease (eosinophilic granuloma), so that the true survival rate for this period may have been higher than Table III would indicate.

Clinical trials almost invariably involve some initial selection of patients depending on the presence or absence of various clinical or laboratory features. Some patients or their parents may refuse consent to be entered into a trial, or an individual case may be judged unsuitable for one reason or another. Additionally, patients may be excluded later from the trial because of relapse, violation of protocol, or an ill-defined reason such as "non-evaluability". A population-based study of survival therefore gives a more realistic assessment of current survival rates and the changes which have occurred over a period of time. The results of this study indicate that the outlook for children with cancer improved considerably around the late 1960s or early 1970s. Relatively little further improvement has occurred in the later 1970s. Over the 25 year period significant improvement occurred in the survival rates of ALL, non-Hodgkin's lymphoma, Hodgkin's disease, Wilms' tumour, medulloblastoma, and retinoblastoma. The results of treatment of some tumours such as neuroblastoma, anaplastic astrocytoma, and brain stem glioma remain poor, while ALL is still an important cause of death in children. There is therefore a need for continuing research into the treatment of childhood cancer, in the hope of producing better results in this latter group. The results of clinical trials in some selected groups of patients should give no cause for complacency.

### References

ANDERSON, J.R., WILSON, J.F., JENKIN, R.D.T., & 8 others. (1983). Childhood non-Hodgkin's lymphoma. *N. Engl. J. Med.*, **308**, 559.

BERRY, M.P., JENKIN, R.T.D., KEEN, C.W., NAIR, B.D. & SIMPSON, W.J. (1981). Radiation treatment for medulloblastoma. *J. Neurosurg.*, **55**, 43.

BIZER, L.S. (1980). Rhabdomyosarcoma. *Am. J. Surg.*, **140**, 687.

BLOOM, H.J.G. (1982*a*). Intracranial tumours: response and resistance to therapeutic endeavors, 1970–1980. *Int. J. Radiat. Oncol. Biol. Phys.*, **8**, 1083.

BLOOM, H.J.G. (1982*b*). Medulloblastoma in children: increasing survival rates and further prospects. *Int. J. Radiat. Oncol. Biol. Phys.*, **8**, 2023.

D'ANGIO, G.J., EVANS, A.E., BRESLOW, N. & 10 others. (1981). The treatment of Wilms' tumor: results of the second national Wilms' tumor study. *Cancer*, **47**, 2302.

DRAPER, G.J., BIRCH, J.M., BITHELL, J.F. & 6 others. (1982). *Childhood Cancer in Britain*. London: H.M.S.O., p. 1.

JENKIN, R.D.T. & BERRY, M.P. (1980). Hodgkin's disease in children. *Semin. Oncol.*, **7**, 202.

McWHIRTER, W.R. & BACON, J.E. (1981). Incidence of childhood tumours in Queensland. *Br. J. Cancer*, **44**, 637.

MYERS, M.H., HEISE, H.W., LI, F.P. & MILLER, R.W. (1975). Trends in cancer survival among U.S. white children, 1955–71. *J. Pediatr.*, **87**, 815.

PETO, R., PIKE, M.C., ARMITAGE, P. & 7 others. (1977). Design and analysis of randomised clinical trials requiring prolonged observation of each patient. *Br. J. Cancer*, **35**, 1.

WILBUR, J.R., KING, O.Y., DE WIT, S.A. & MOTT, M.G. (1981). Non-lymphoblastic leukaemia in children: long term survival with chemotherapy. *Proc. Soc. Clin. Oncol.*, **22**, 482.

WOLLNER, N., BURCHENAL, J.H., LIEBERMAN, P.H., EXELBY, P., D'ANGIO, G. & MURPHY, M.L. (1976). Non-Hodgkin's lymphoma in children. *Cancer*, **37**, 123.


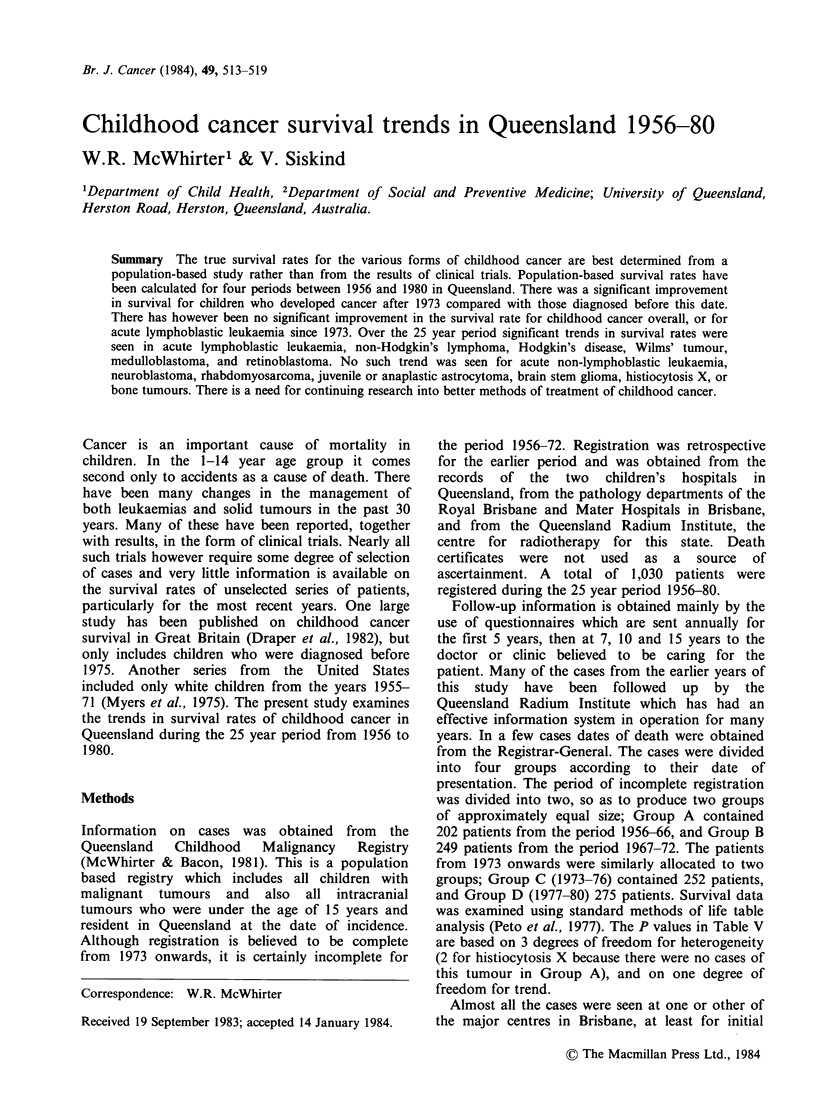

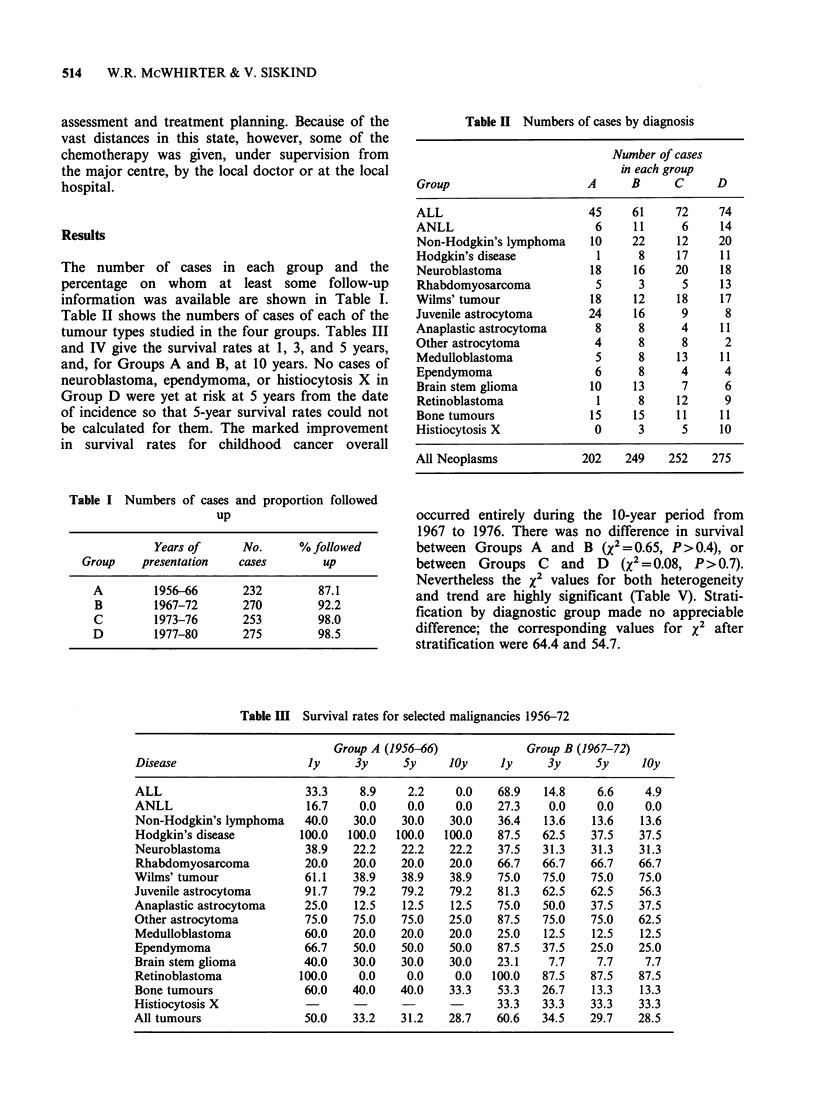

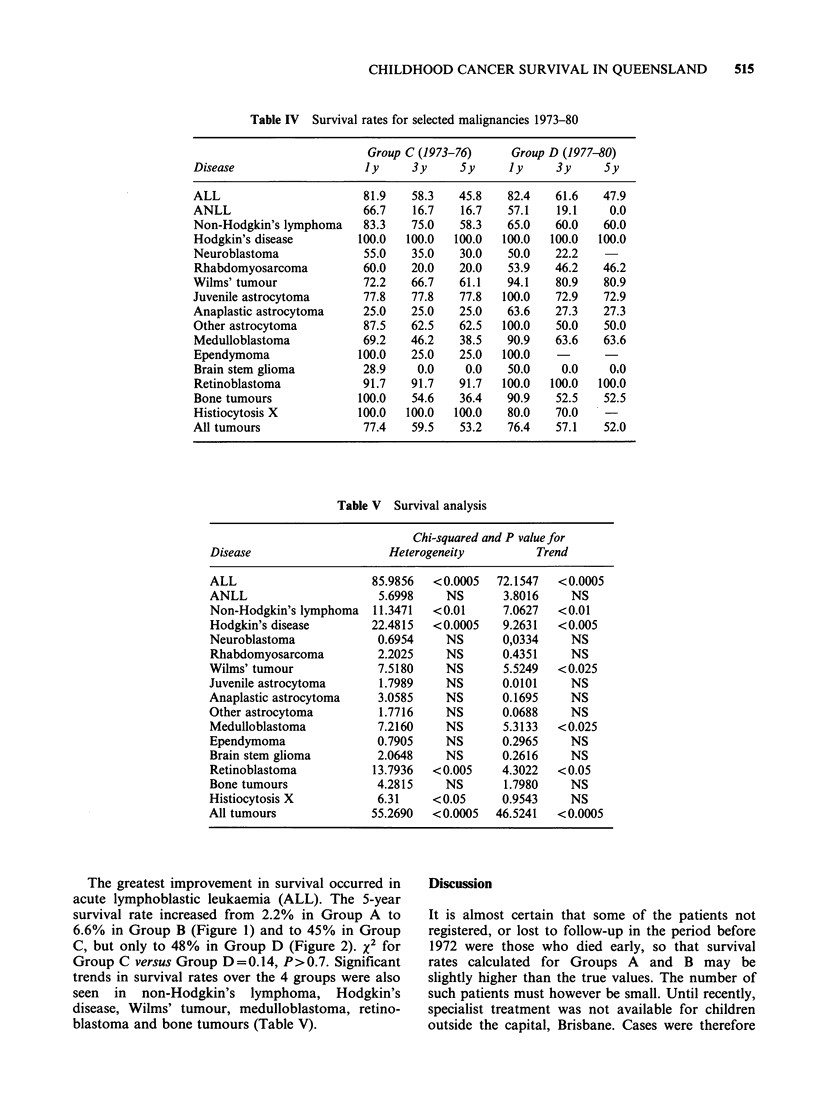

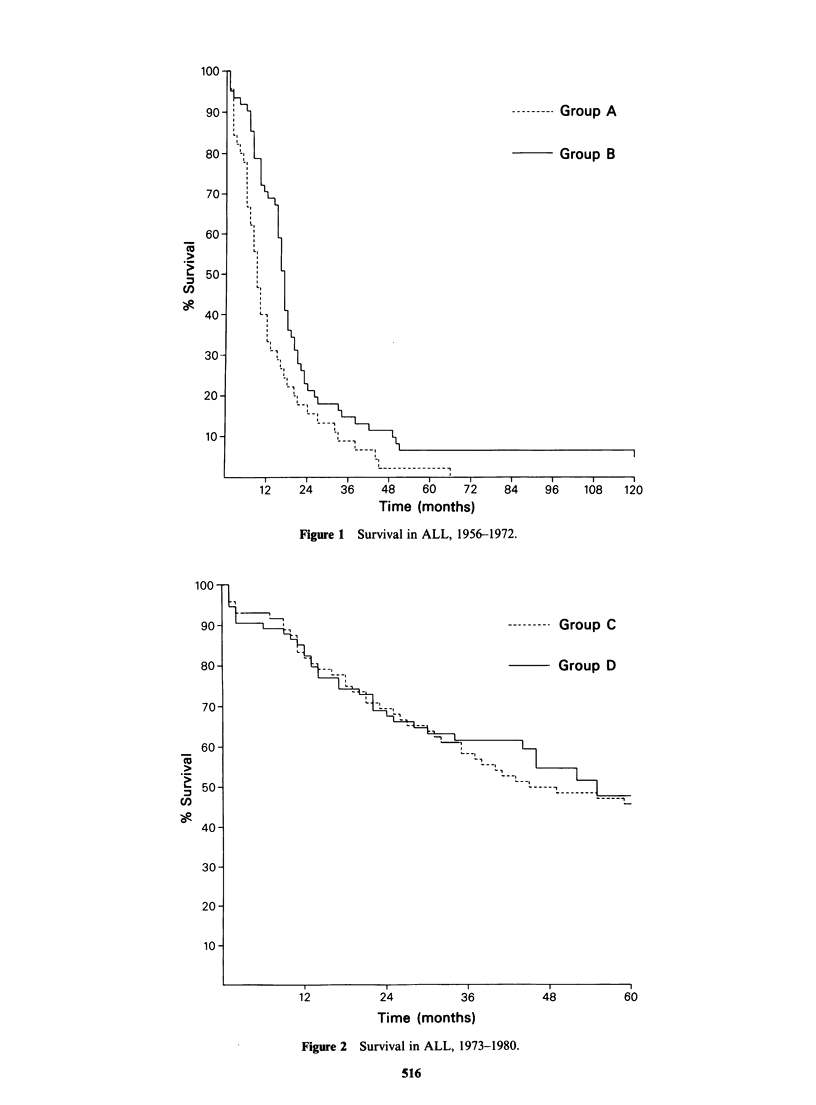

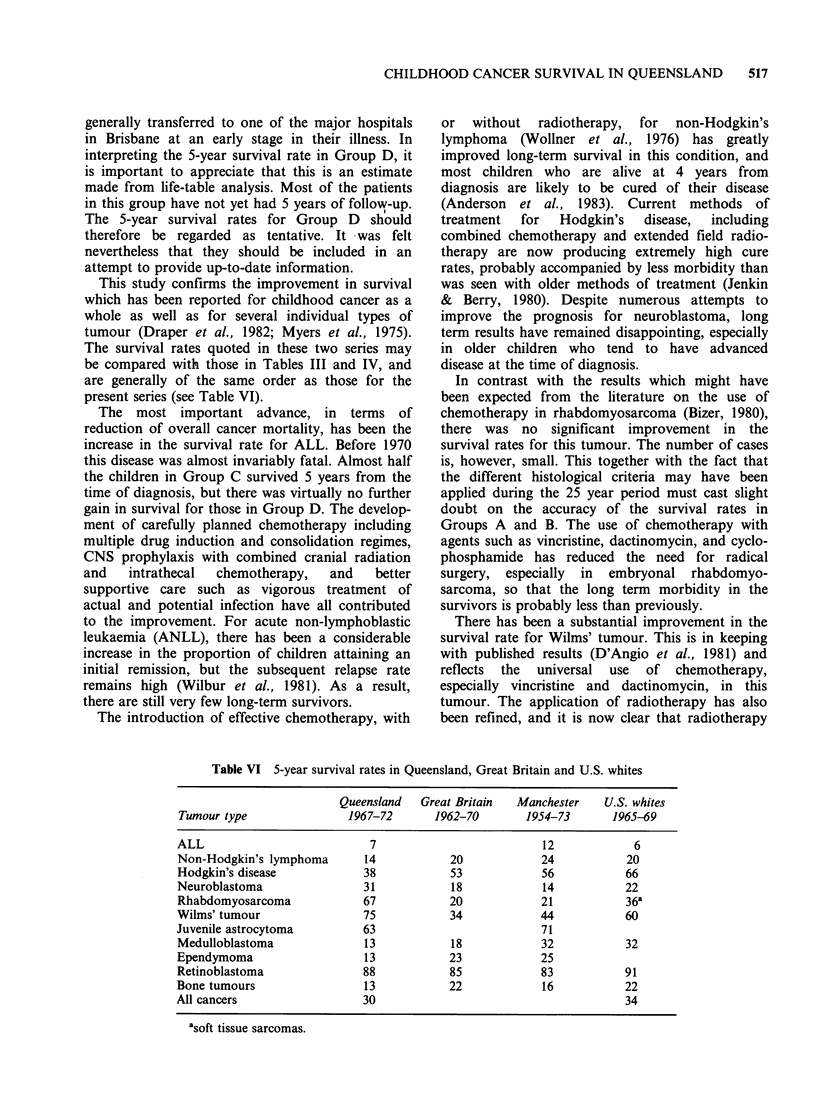

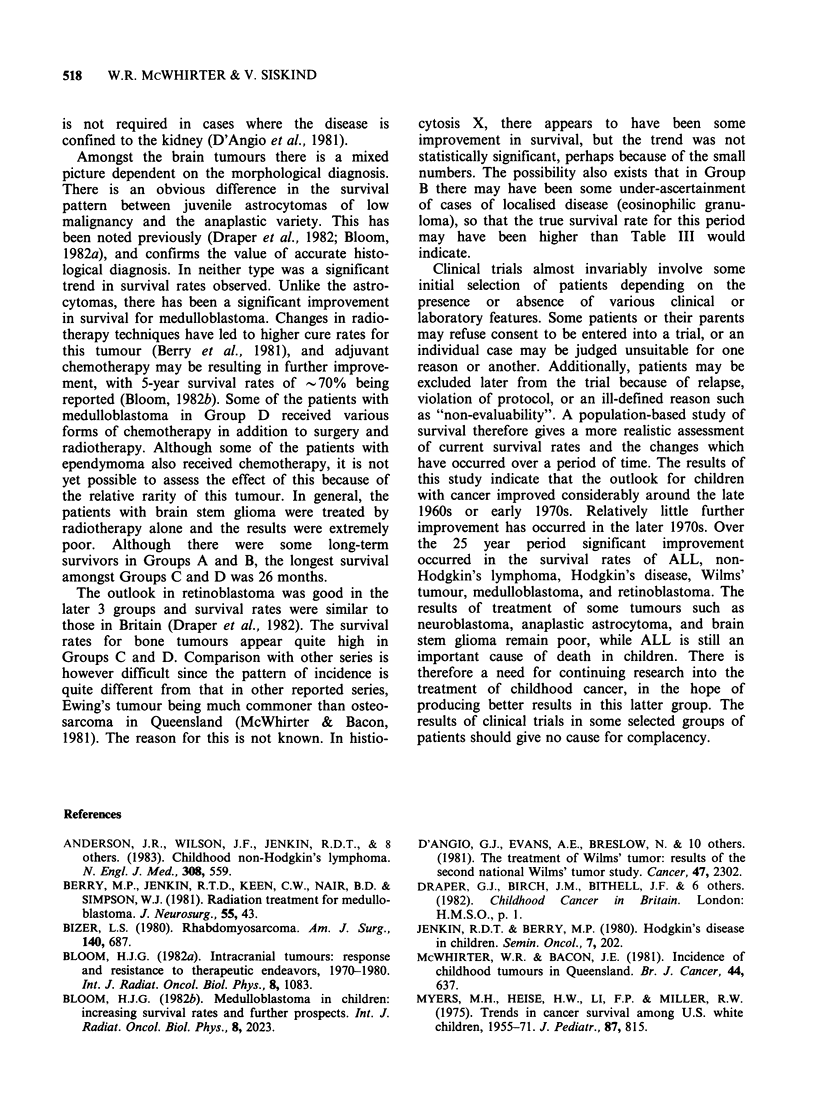

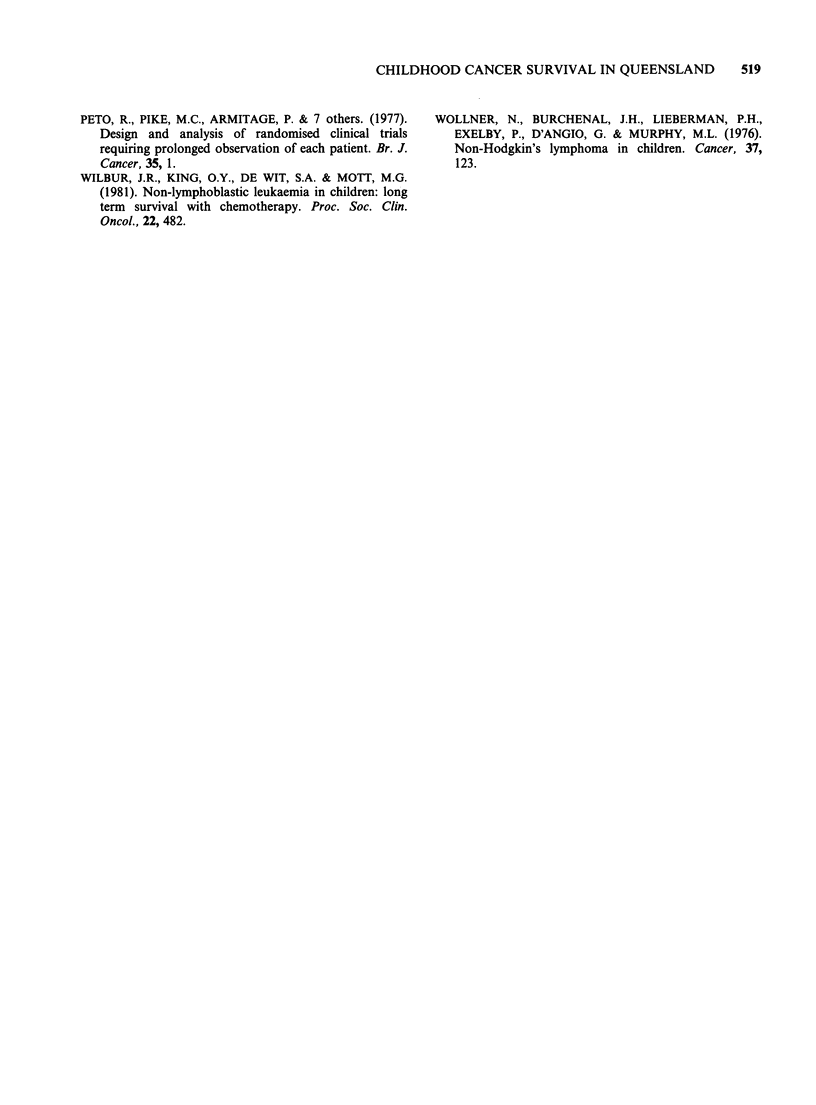


## References

[OCR_00614] Anderson J. R., Wilson J. F., Jenkin D. T., Meadows A. T., Kersey J., Chilcote R. R., Coccia P., Exelby P., Kushner J., Siegel S. (1983). Childhood non-Hodgkin's lymphoma. The results of a randomized therapeutic trial comparing a 4-drug regimen (COMP) with a 10-drug regimen (LSA2-L2).. N Engl J Med.

[OCR_00619] Berry M. P., Jenkin R. D., Keen C. W., Nair B. D., Simpson W. J. (1981). Radiation treatment for medulloblastoma. A 21-year review.. J Neurosurg.

[OCR_00624] Bizer L. S. (1980). Rhabdomyosarcoma.. Am J Surg.

[OCR_00628] Bloom H. J. (1982). Intracranial tumors: response and resistance to therapeutic endeavors, 1970-1980.. Int J Radiat Oncol Biol Phys.

[OCR_00633] Bloom H. J. (1982). Medulloblastoma in children: increasing survival rates and further prospects.. Int J Radiat Oncol Biol Phys.

[OCR_00638] D'Angio G. J., Evans A., Breslow N., Beckwith B., Bishop H., Farewell V., Goodwin W., Leape L., Palmer N., Sinks L. (1981). The treatment of Wilms' tumor: results of the Second National Wilms' Tumor Study.. Cancer.

[OCR_00647] Derek R., Jenkin T., Berry M. P. (1980). Hodgkin's disease in children.. Semin Oncol.

[OCR_00651] McWhirter W. R., Bacon J. E. (1981). Incidence of childhood tumours in Queensland.. Br J Cancer.

[OCR_00656] Myers M. H., Heise H. W., Li F. P., Miller R. W. (1975). Trends in cancer survival among U.S. white children, 1955-1971.. J Pediatr.

[OCR_00675] Wollner N., Burchenal J. H., Lieberman P. H., Exelby P., D'Angio G., Murphy M. L. (1976). Non-Hodgkin's lymphoma in children. A comparative study of two modalities of therapy.. Cancer.

